# Case Report: Giant uterine broad ligament fibroid

**DOI:** 10.3389/fonc.2025.1712549

**Published:** 2026-01-07

**Authors:** Weiwei Qian, Shaoqin Sheng, Xiangqian Xu, Jiaxin Miao, Yanying Nong

**Affiliations:** 1 The Fourth Clinical Medical College of Zhejiang Chinese Medical University, Hangzhou, China; 2 Department of Gynecology, Hangzhou Obstetrics and Gynecology Hospital, Hangzhou, China; 3 Clinical Medical College of Hangzhou Normal University, Hangzhou, China

**Keywords:** case report, diagnosis and treatment strategies, giant uterine fibroids, intraoperative myomectomy during cesarean section, surgical techniques

## Abstract

Giant uterine fibroids that arise shortly after cesarean section are a rare and distinctive clinical entity. In this research, a retrospective analysis was carried out on the clinical data of a particular patient with such giant uterine fibroids who was admitted to Hangzhou Obstetrics and Gynecology Hospital. What made this case remarkable was that the patient quickly developed symptoms of pelvic and abdominal compression in the period following cesarean section. Imaging examinations disclosed a giant tumor located in the right broad ligament area. Moreover, there was extensive adhesion among the surrounding tissues and the anatomical structure was in disarray, which underlined the complexity of the condition. During the surgical treatment process, a combination of sharp separation and blunt dissection techniques was utilized. In addition, precise management of blood vessels and strategies for protecting important organs were also implemented. Ultimately, the tumor was successfully and completely removed. Postoperative pathology results confirmed that it was a uterine leiomyoma with edematous degeneration. The patient recovered smoothly after the surgery without experiencing any serious complications. Furthermore, no recurrence of the tumor was noticed during the follow-up period. In general, given the rarity, rapid progression, and intricate anatomical connections of giant uterine fibroids after cesarean section, it is of crucial importance to conduct preoperative multimodal imaging evaluations. These evaluations can help clarify the scope of the tumor as well as its adjacent relationships. Besides, during the operation, attaching great importance to adhesion separation techniques and taking good care of important blood vessels and organs, along with the implementation of individualized surgical plans, can remarkably improve the safety and effectiveness of the treatment for these tough and uncommon cases. Meanwhile, taking the clinical diagnosis and management of pregnancy complicated with giant broad ligament fibroids as an entry point, this study systematically demonstrates the clinical advantages and technical feasibility of concurrent myomectomy during cesarean section through the analysis of surgical strategies, operational details, and long-term follow-up data of a typical case. It is intended to provide evidence-based references for individualized clinical decision-making in similar cases.

## 1 Introduction

Uterine fibroids are the most common benign tumors in the female reproductive system, with an incidence rate ranging from 5.4% to 77% among women of childbearing age (1). In most cases, uterine fibroids grow slowly and the symptoms are latent, having a relatively minor impact on patients’ quality of life. However, rare giant uterine fibroids, as a special type of uterine fibroids, can experience an abnormally rapid increase in tumor volume within a short period of time, causing significant compression on surrounding tissues and organs and leading to a series of severe symptoms.

Here, we present a case report detailing the clinical diagnosis and management of a patient with a giant uterine fibroid, admitted to Hangzhou Women’s Hospital. Notably, the fibroid achieved a maximum diameter of 25 cm shortly following the patient’s cesarean section. The following sections outline the case specifics in detail.

## 2 Case presentation

**Figure 1:**
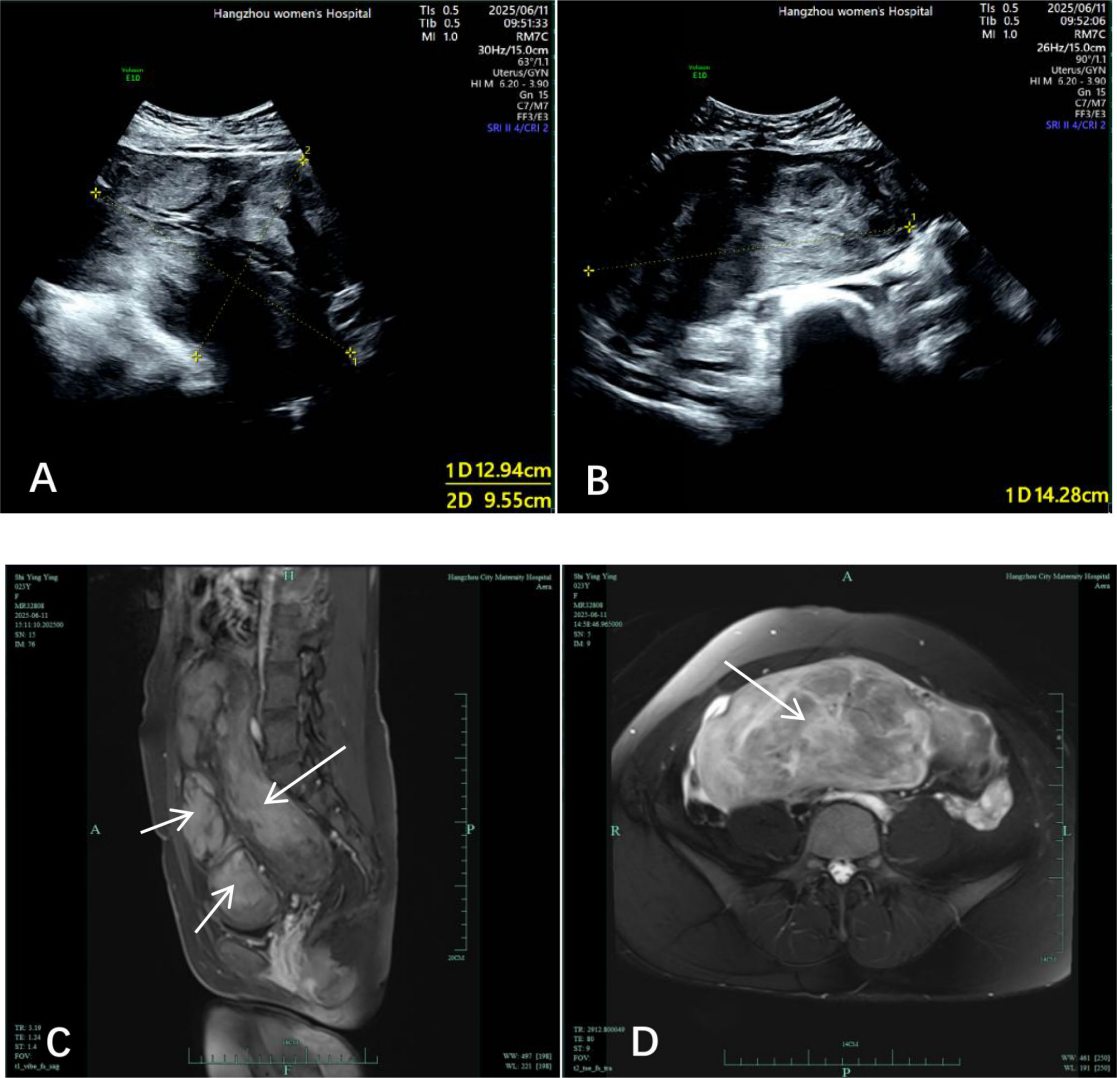
Preoperative transvaginal gynecological ultrasound of the patient revealed a massive abdominopelvic mass, which was considered to be of uterine origin with undetermined nature **(A, B)**. Preoperative MRI revealed a massive mass shadow in the abdominopelvic cavity. On T1-weighted images (T1WI), the arrows indicate that the mass has relatively clear boundaries and exhibits lobulated margins **(C)** on T2-weighted images (T2WI), the arrows show that the mass fills the entire abdominopelvic cavity, adheres closely to the pelvic wall, and compresses the surrounding organs **(D)**.

A 23-year-old married female (gravida 1, para 1) was admitted to our hospital on June 11, 2025, with a 18-month history of uterine fibroids and 1-week duration of abdominal distension. Eighteen months prior, during the third trimester of her pregnancy, two uterine fibroids were detected by ultrasound, with the larger one measuring 10 cm in diameter, and she underwent a lower uterine segment cesarean section at a local hospital on February 13, 2024, during which a fibroid-like protrusion approximately 10 cm in size was observed on the right posterior side of the uterus without any intervention; her first post-operative menstrual period resumed on August 24, 2024, and since this menstrual period, her menstrual flow has been significantly heavier than that before pregnancy, yet she did not attend regular follow-up visits, with her last menstrual period being on May 21, 2025; one week prior to admission, the patient developed an unprovoked sensation of abdominal fullness, accompanied by constipation, anal distension discomfort, as well as frequent urination and urgent urination, and occasional dizziness and fatigue, thus she sought medical attention at our hospital; color Doppler ultrasound at our hospital indicated a huge abdominopelvic mass, which was considered to be of uterine origin with undetermined nature (the mass measured approximately 22.8×9.6×14.3 cm, with clear boundaries and a lobulated shape), and she was admitted to the hospital via the outpatient department for further diagnosis and treatment. On admission physical examination, the patient was alert with stable mental status, her blood pressure was 100/68 mmHg (1 mmHg = 0.133 kPa), pulse rate was 80 beats per minute, and conjunctival pallor was noted; her abdomen was distended and globular, with a transverse surgical scar approximately 12 cm in length in the midline of the lower abdomen, the abdomen was soft, and a huge mass was palpable from above the symphysis pubis to below the xiphoid process, extending to the right midaxillary line, which was hard in texture, relatively smooth on the surface, and had poor mobility. Gynecological examination showed a nulliparous vulvar configuration, a patent vagina with a small amount of white discharge, mild cervical hypertrophy with erosion, and narrow fornix space, the uterine fundus was located 2 finger-breadths below the xiphoid process, with an abnormal shape, hard texture, poor mobility, and no tenderness, and no obvious abnormalities were palpable in the bilateral adnexal regions. Laboratory tests revealed that the hemoglobin level in the complete blood count was 81 g/L, while no significant abnormalities were found in liver and kidney function, four coagulation parameters, five thyroid function indicators, and female tumor markers. Transvaginal gynecological color Doppler ultrasound showed that the uterus was in an anterior position, with a double-layer endometrial thickness of 0.9 cm and a cervical length of approximately 8.0 cm, and a huge mass was visible on the right side of the uterus and cervix, measuring approximately 22.8×9.6×14.3 cm, with clear boundaries and a lobulated shape, color Doppler flow imaging (CDFI) showed blood flow signals inside the mass, with a resistive index (RI) of 0.50 (**Figures 1A, B**), and the mass was considered a huge abdominopelvic mass, suspected to be of uterine origin with undetermined nature; pelvic magnetic resonance imaging (MRI) plain scan plus contrast enhancement showed a huge mass shadow on the right side of the pelvic cavity, which presented isointensity on T1-weighted images (T1WI) and heterogeneous intensity on T2-weighted images (T2WI), with relatively clear boundaries and lobulated edges, no diffusion restriction was observed on diffusion-weighted imaging (DWI), gradual heterogeneous enhancement was seen on contrast-enhanced scanning, the mass measured approximately 21.6×14.0×12.3 cm, the lesion was closely related to the right lateral wall of the uterus, with locally thickened feeding artery shadows visible, no abnormalities were found in the signal, size, and shape of the bilateral ovaries, no obvious enlargement of pelvic lymph nodes was observed, and a small amount of free fluid was present in the pelvic cavity (**Figures 1C, D**); ultrasound of the female urinary system, liver, kidneys, spleen, and pancreas showed no significant abnormalities.

## 3 Diagnostic assessment and outcome

**Figure 2:**
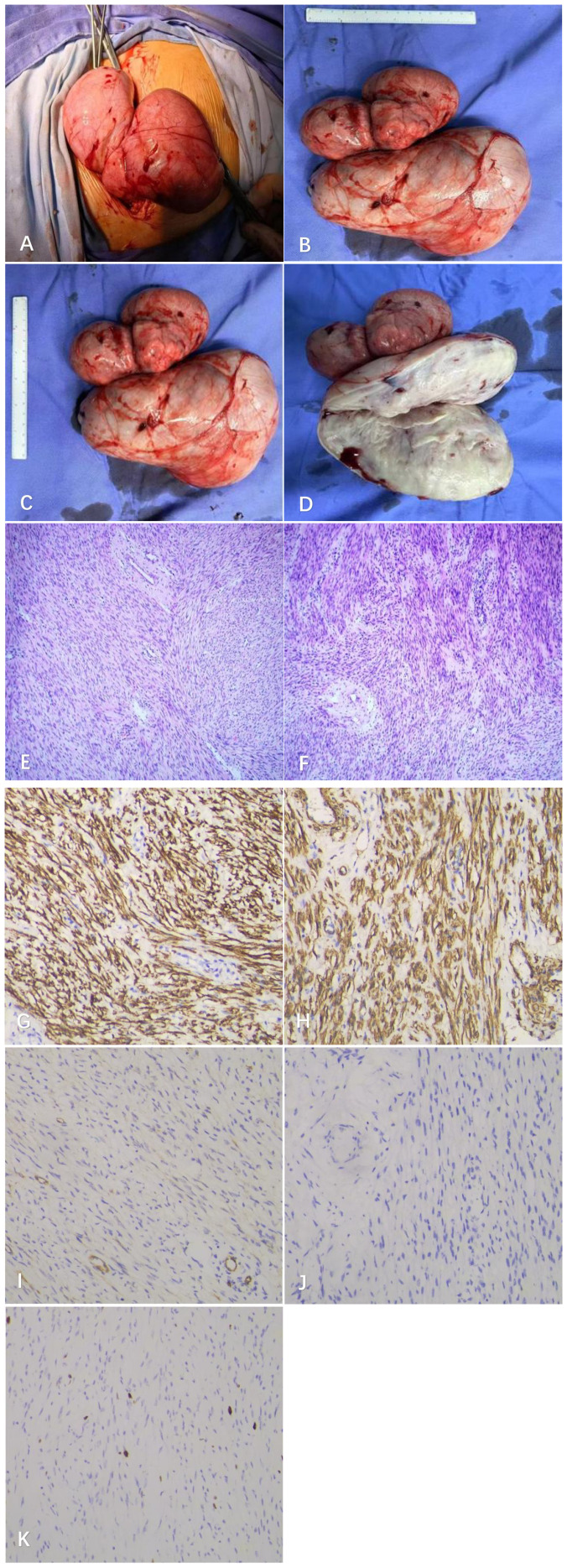
The gross morphology of the tumor during the operation **(A-D)**. The postoperative pathological results of the tumor indicated a uterine spindle cell tumor. HE-stained sections at a magnification of 200× **(E, F)**. The results of immunohistochemical (IHC) staining, Desmin+ **(G)**, SMA+ **(H)**, CD10- **(I)**, ALK- **(J)**, Ki-67+ **(K)**, all sections were examined at a magnification of 200×.

Our department, in collaboration with the Department of Urology, Department of Anesthesiology, Department of Cardiovascular Medicine, and other relevant departments, systematically reviewed and comprehensively evaluated the patient’s clinical data, and performed open broad ligament tumor resection and intestinal adhesion lysis under general anesthesia on June 12, 2025. Given the high anatomical overlap between the ureteral course and the broad ligament region, and considering that long-term compression by the fibroid in this case may have caused ureteral anatomical displacement, we performed preoperative right ureteral double-J stent placement to mitigate this risk at its source. Specifically, the distal end of the double-J stent was fixed in the urinary bladder, and the proximal end was placed in the renal pelvis under cystoscopy. Leveraging the supportive and drainage functions of the double-J stent, this intervention served two key purposes: first, it allowed for precise intraoperative localization of the ureteral course by palpating the stent, thereby clarifying its spatial relationship with the tumor and adherent tissues; second, even in the event of minor iatrogenic injury during surgery, the double-J stent could ensure unobstructed urinary drainage and reduce the incidence of severe complications such as urinary extravasation. The surgery was conducted through the previous cesarean section incision, and intraoperative exploration revealed that the uterus was enlarged to the size of a 2-month pregnancy with a significantly abnormal shape, which was markedly displaced to the left and closely adhered to the left pelvic wall due to compression, resulting in the loss of its normal anatomical position, a massive neoplastic lesion was detected in the right broad ligament, with dimensions of approximately 25.0 cm × 10.0 cm × 16.0 cm, presenting a multilocular and lobulated appearance, and the texture of local areas was relatively soft accompanied by obvious edematous degeneration. Due to its huge size, the tumor had completely occupied the abdominopelvic space and formed extensive and dense adhesions with the right abdominopelvic wall and parts of the intestinal tract, making anatomical spaces difficult to identify; the pouch of Douglas was severely deformed due to continuous compression by the tumor; the bilateral parauterine blood vessels showed significant tortuosity and distension, indicating local hemodynamic abnormalities; the right fallopian tube was compressed by the tumor and densely adhered to the uterine wall, with its lumen shape unidentifiable, while no obvious abnormalities were found in the shape and structure of the left fallopian tube and bilateral ovaries. Guided by the localization of the double-J stent, we first approached the loosely adherent areas and gradually released adhesions between the tumor and the pelvic abdominal wall as well as intestinal loops using a combination of sharp and blunt dissection. Fine dissection was performed along the potential space between the tumor and adjacent structures (pelvic abdominal wall and intestinal loops) with an ultrasonic scalpel. Throughout the procedure, continuous identification of the boundary between the intestinal serosa and the superficial fascia of the broad ligament was essential to avoid injury to adjacent organs. When approaching the region of the ureteral course, the position of the double-J stent was confirmed by gentle palpation. Dissection of the tumor capsule was strictly performed along the lateral margin of the ureter to prevent ureteral wall injury or transection caused by excessive traction. Meanwhile, real-time intraoperative observation of the double-J stent for displacement or pulsation was used to indirectly assess the ureteral blood supply and functional status. During the dissection, active bleeding occurred on multiple occasions, and repeated hemostasis with hemostats was performed to expose the right broad ligament region. after injecting 6 units of pituitrin into the uterine myometrium, the anterior leaf of the broad ligament was incised to gradually expose the capsule on the surface of the tumor, and blunt dissection was carried out along the space, with close adherence to the tumor capsule during the process to avoid damage to important structures such as the ureter and iliac blood vessels. Since the tumor occupied the entire abdominopelvic cavity, the intraoperative operating space was extremely limited, and finally, the tumor was completely dissected and removed after a procedure lasting 3 hours and 20 minutes. The preoperatively placed right ureteral double-J stent was removed immediately upon completion of the surgery. The surgery proceeded smoothly, with one pelvic drainage tube indwelled, intraoperative urine output of 50 ml, and blood loss of approximately 700 ml, for which 2 units of suspended red blood cells and 200 ml of plasma were transfused. The gross appearance of the tumor resected during the operation is shown in **Figures 2A–D**. The postoperative pathological report indicated a (uterine) spindle cell tumor, which was consistent with leiomyoma with focal edema based on morphology and immunohistochemistry; H&E staining of the uterine fibroid is shown in **Figures 2E, F**; the immunohistochemical (IHC) results of the leiomyoma were as follows: Desmin (+), SMA (+), CD10 (−), ALK (−), and Ki67 (+, 8%) (**Figures 2G–K**).

**Figure 3:**
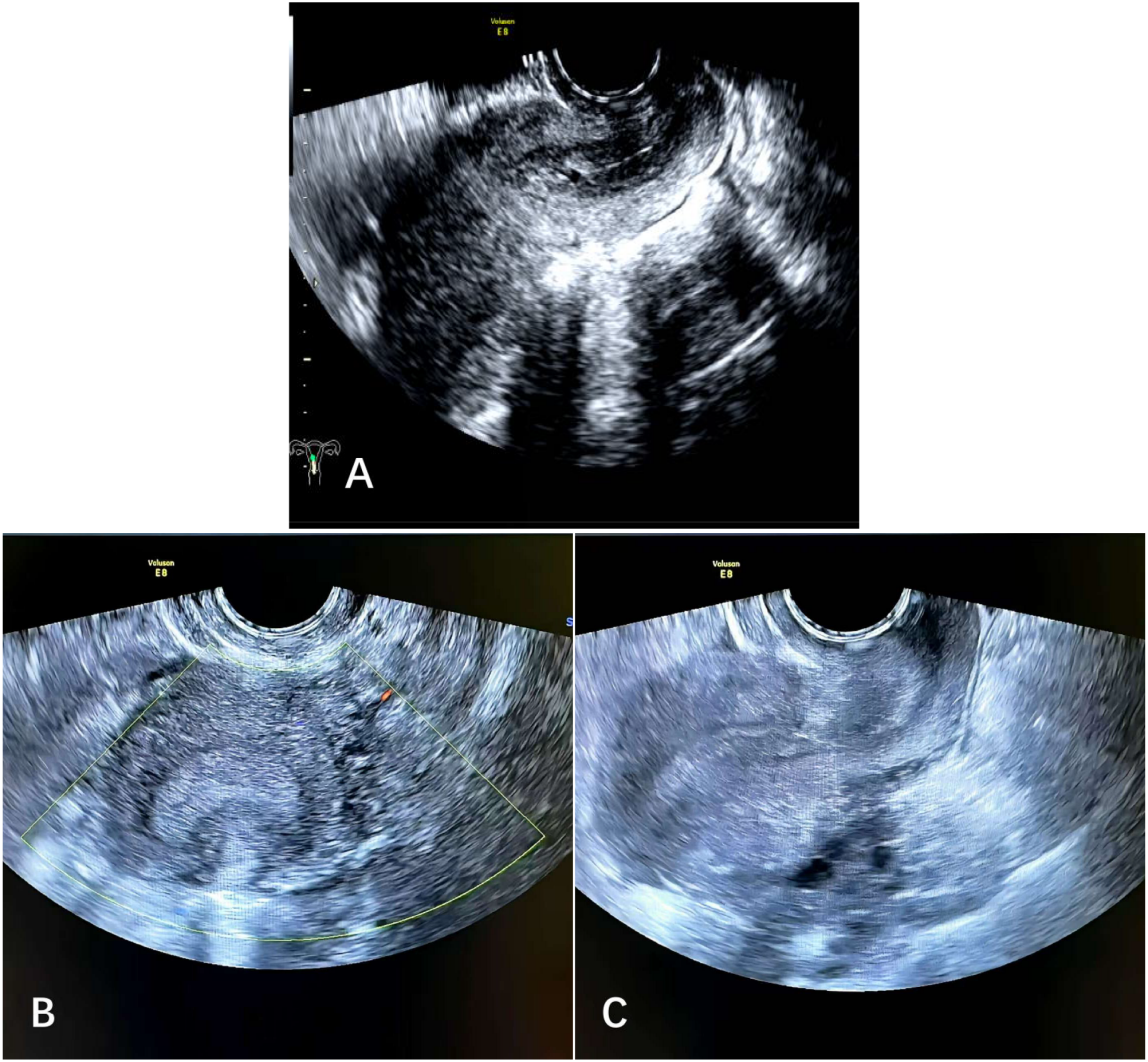
Transvaginal B-ultrasound re-examination one month after surgery showed no obvious abnormalities in the uterus and bilateral adnexa **(A)**. Transvaginal B-ultrasound re-examination five months after surgery showed no obvious abnormalities in the uterus and bilateral adnexa **(B, C)**.

Postoperative laboratory tests showed a hemoglobin level of 59 g/L, a white blood cell count of 11.8×10^9/L, and a high-sensitivity C-reactive protein level of 22.09 mg/L. The patient was given symptomatic treatments including oxytocin to promote uterine contraction, antibiotics for infection prevention, and fluid replacement support. The pelvic drainage tube was removed on the 2nd postoperative day, and the patient was discharged from the hospital on the 7th postoperative day. One month after the operation, the patient returned to the gynecological outpatient department of our hospital for a follow-up visit, examinations showed that the surgical incision had healed well, and no obvious abnormalities were found in the transvaginal gynecological color Doppler ultrasound (**Figure 3A**). Five months after surgery, the patient returned to our hospital for a follow-up examination. Gynecological examination revealed no abnormalities, and laboratory tests (including routine blood tests, biochemical indicators, and tumor markers) were all within the normal range. Additionally, imaging examination (transvaginal gynecological color Doppler ultrasound) showed no significant abnormalities (**Figures 3B, C**).

## 4 Discussion

Giant uterine fibroids are relatively rare in clinical practice. Internationally, they generally refer to uterine fibroids exceeding 11.4 kg (25 pounds) (2), while domestically, they are typically defined as single fibroids with a diameter greater than 12 cm or a size larger than that of a 6-week pregnancy (3). Due to their significantly increased volume, giant uterine fibroids often break through the original space limitations of the pelvic cavity, protrude out of the pelvis, and may compress surrounding organs (4). When the fibroid compresses the bladder, patients may experience bladder irritation symptoms such as frequent urination and urgent urination, and in severe cases, even dysuria; when it compresses the rectum, patients mostly present with constipation and difficulty in defecation; if it compresses the ureter, urinary drainage is obstructed, which can gradually lead to hydronephrosis and subsequent low back pain (5). Meanwhile, the abnormal enlargement of the uterine volume caused by fibroid growth leads to a corresponding increase in the endometrial area, so patients may experience symptoms such as significantly increased menstrual flow compared with the normal state, prolonged menstrual periods, and menstrual cycle disorders, long-term increased menstrual flow can further cause secondary anemia, manifested as systemic symptoms including pale complexion, fatigue, and dizziness (6).

**Figure 4:**
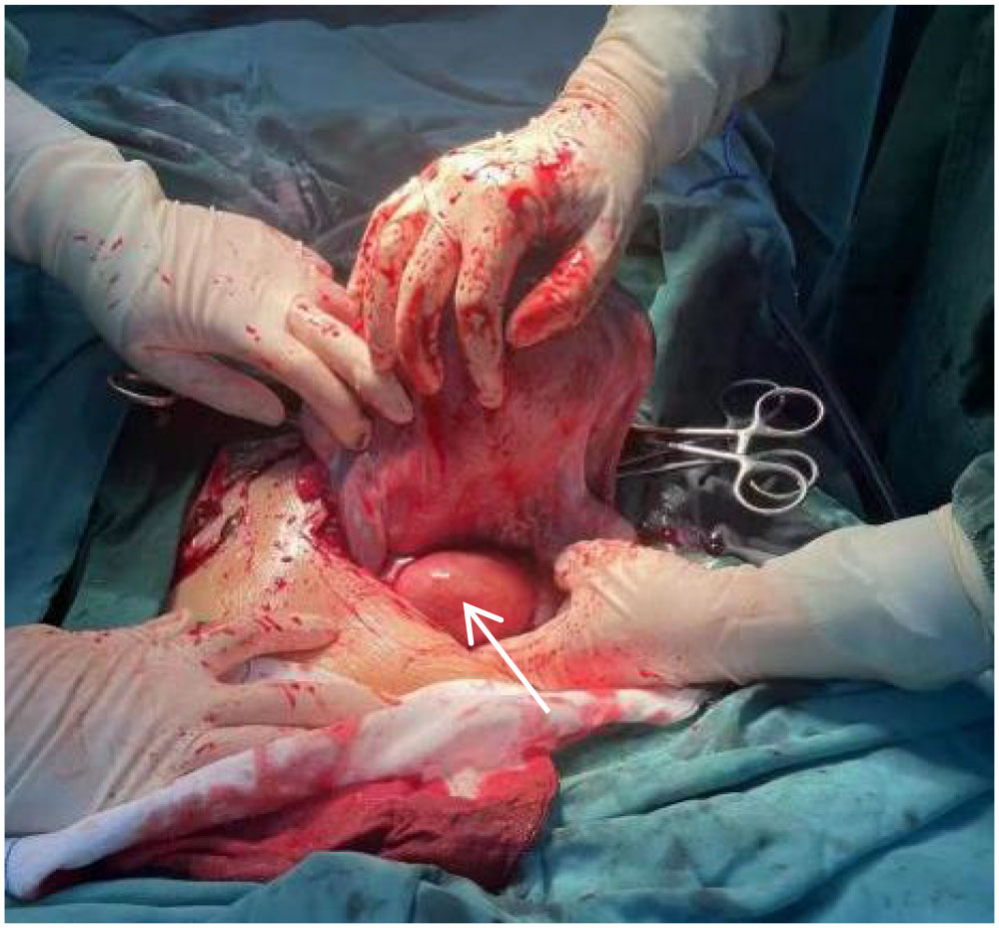
The uterine fibroid that was not managed during the patient’s cesarean section (indicated by the arrow).

In recent years, the incidence of uterine fibroids complicating pregnancy has shown an upward trend. During pregnancy, the significant elevation of estrogen and progesterone levels in pregnant women can induce rapid growth of uterine fibroids, which exerts adverse effects on maternal and fetal outcomes (7). For uterine fibroids detected during pregnancy, conservative treatment is typically the primary approach to prevent complications such as preterm labor and miscarriage; for parturients with pregnancy complicated by giant uterine fibroids, cesarean section is often a more appropriate option for terminating pregnancy when selecting the mode of delivery (8). However, there remains clinical controversy regarding whether to perform concurrent myomectomy during cesarean section (9). The core lies in balancing the potential benefits and inherent risks of the procedure, while making individualized judgments based on the patientts specific clinical profile. Regarding the potential advantages, concurrent surgery can spare patients from secondary surgical trauma due to residual fibroids postpartum, reducing medical costs associated with anesthesia, hospitalization, and postoperative rehabilitation. Additionally, timely myomectomy can eliminate the adverse impact of fibroids on postpartum uterine involution and mitigate the risk of rapid fibroid enlargement, degeneration, or malignant transformation in the short term. Particularly for large-sized fibroids (e.g., 10 cm in diameter preoperatively as in this case), it can prevent subsequent complications such as abdominal distension and constipation caused by compression of adjacent organs, thereby improving the patient’s postpartum quality of life. Furthermore, concurrent surgery alleviates the economic and time burden of long-term follow-up and monitoring for patients, while avoiding increased surgical complexity and elevated risks arising from fibroid progression. However, the potential risks of concurrent myomectomy during cesarean section should not be underestimated. The uterus exhibits abundant blood supply during pregnancy, and fibroid tissue is often hyperemic, edematous, and fragile. Especially for large fibroids or those located intramurally or submucosally, excessive bleeding from the surgical site and difficulties in hemostasis are prone to occur during myomectomy. This may lead to intraoperative blood transfusion, postoperative hemorrhage, or even massive hemorrhage, and hysterectomy may be necessitated in severe cases (10). Meanwhile, increased surgical trauma can compromise the integrity of the uterine myometrium, impair postoperative uterine contractile function, prolong lochia discharge, and elevate the risk of complications such as intrauterine infection and pelvic adhesion. In extreme cases, it may also induce coagulation disorders, endangering the patientri life safety. Additionally, if the patient presents with uterine atony following fetal delivery or unstable vital signs during surgery, concurrent myomectomy will further exacerbate the systemic stress response and increase surgical uncertainty. Due to potential phasic changes in estrogen and progesterone following their rapid decline postpartum, some fibroids may develop congestion, edema, and other changes that lead to a short-term increase in volume, causing compressive symptoms such as abdominal distension, frequent urination, constipation, and dysuria. An increasing body of research (11–14) has indicated that performing myomectomy concurrently with cesarean section does not increase intraoperative injury in patients; with adequate preoperative preparation and careful surgical manipulation based on factors such as the location, number, and size of fibroids, concurrent myomectomy during cesarean section is a reasonable and safe surgical modality. The patient in this case resides in a rural area of Henan Province, China. Due to relatively underdeveloped economic conditions, she never underwent specialized gynecological examinations at a hospital before or during the first trimester of pregnancy. It was not until the third trimester that uterine fibroids were first detected via ultrasound, with the largest measuring 10 cm in diameter. Additionally, the patient had no definite prior history of uterine fibroids and no family history of related diseases. On February 13, 2024, she underwent cesarean section to terminate pregnancy due to pregnancy complicated by uterine fibroids, and the 10-cm fibroid was not managed during the operation (**Figure 4**), nor was postoperative follow-up conducted, as reported, the operating surgeon determined that the fibroid did not exert an urgent adverse impact on fetal delivery. Given that concurrent enucleation of a 10-cm fibroid during cesarean section would significantly increase the risks of intraoperative hemostatic difficulties and postpartum hemorrhage, no intervention was performed during the surgery, and no postoperative follow-up was conducted. In the context of the clinical background, the patient is a resident of Lfsident Village, Neixiang County, Nanyang City, Henan Province, China. Due to the relatively disadvantaged economic status of her family, she had never undergone specialized gynecological examinations prior to pregnancy, nor did she receive standardized prenatal care during the first trimester. Additionally, she failed to complete the routine 42-day postpartum follow-up as recommended in China. Throughout the diagnosis and treatment process, the patient maintained a stable psychological state with no history of mental health conditions such as anxiety or depression. She did not refuse concurrent myomectomy during surgery or postoperative follow-up due to excessive concerns about surgical risks or cognitive misunderstandings. The patient presented to our hospital on this occasion with clinical symptoms including significantly increased menstrual flow, marked abdominal distension, and constipation after childbirth. The examinations revealed that the original uterine fibroid had rapidly grown to approximately 25 cm in diameter within a short period, substantially increasing the risk of reoperation.

The overall incidence of uterine fibroid malignant transformation is relatively low (approximately 0.4%–0.8%) (15). However, the special physiological state after childbirth may increase monitoring difficulty or potential risks, and the uterus itself is in a recovery stage postpartum, which may present with manifestations such as uterine enlargement, abdominal pain, and vaginal bleeding—symptoms similar to the early signs of fibroid malignant transformation (e.g., rapid fibroid growth), leading to easy confusion and delayed diagnosis. In this case, the fibroid volume increased rapidly within 16 months after the patient’s childbirth, and imaging findings suggested that low-grade malignant tumors could not be completely ruled out. The postoperative routine pathological diagnosis was uterine spindle cell tumor (morphologically first considered leiomyoma with focal edema), and immunohistochemical detection showed SMA (+), Desmin (+), and Ki67 (+, 8%). The SMA (Smooth Muscle Actin) and Desmin are both myogenic markers, which can strongly confirm the smooth muscle origin of the tumor cells. Ki67 is a nuclear antigen associated with cell proliferation, expressed only in the cell nuclei of proliferating cells, its positive rate (Ki67 index) can reflect the proliferative activity of cells. The patient’s 8% positive rate is consistent with the proliferative characteristics of benign tumors, indicating a relatively low risk of tumor malignant transformation and a relatively good prognosis.

Giant uterine fibroids are already rare cases in clinical practice, and single fibroids with a diameter of 25 cm account for only a very small proportion of these. Notably, there remains a lack of unified and authoritative epidemiological data regarding the incidence of giant uterine fibroids in the current oncology field, with the core constraint being the absence of a consensus on the diagnostic criteria for this condition. Such ambiguity in diagnosis is predominantly reflected in the significant discrepancies between relevant definitions at home and abroad. In the context of this patient, the rapid growth of a giant uterine fibroid from 10 cm to 25 cm in a short period is even more rare. A synthesis of multiple clinical practices and case reports indicates that surgical treatment remains the currently recognized first-choice intervention for patients with giant uterine fibroids, with surgical approaches including laparoscopic surgery and open surgery (16). For patients with no fertility needs, total hysterectomy can completely cure fibroids and prevent recurrence; for patients who wish to preserve the uterus, myomectomy can not only retain the uterus but also significantly reduce the fibroid volume and improve compressive symptoms. The choice between open surgery and laparoscopic surgery requires comprehensive consideration of factors such as the location, number, and size of the fibroids as well as the patient’s own wishes. In this case, the surgical approach was chosen to enter through the midline abdominal incision from the previous cesarean section, combined with preoperative imaging findings indicating a massive abdominopelvic mass with extensive and tight adhesions to surrounding tissues, and important anatomical structures such as iliac blood vessels and ureters being adjacent to the tumor, there was a high possibility of dense adhesions during the operation, leading to severe limitation of the operating space in the abdominopelvic cavity and extremely high difficulty in performing laparoscopic surgery, thus making open surgery the appropriate choice. At the same time, considering the patient’s young age and her wish to preserve fertility, open right broad ligament myomectomy plus intestinal adhesion lysis was planned after comprehensive evaluation. In the surgical management of broad ligament fibroids, the identification and dissection of the ureter constitute a core component in ensuring surgical safety. Given the specific clinical characteristics of the patient in this case, we implemented a targeted perioperative protective measure, namely the placement of a right ureteral double-J stent. During the operation, the adhesions between the tumor and the abdominopelvic wall as well as the intestinal tract were distributed in a carpet-like pattern, and the adhesion interface had lost the normal tissue layer due to chronic inflammatory reactions, during the dissection process, it was necessary not only to avoid injury to the intestinal tract but also to prevent damage or even rupture of adjacent organs around the tumor (such as the ureter and iliac blood vessels) caused by excessive traction—these organs had undergone structural displacement due to long-term compression by the tumor. By dynamically matching the real-time surgical field with preoperative imaging data, repeatedly verifying the anatomical location of tissues during the operation, and combining with accurate judgment of tissue characteristics, the surgeons finally completed this high-difficulty operation in an extremely limited safe space while avoiding injury to adjacent organs, providing valuable practical experience for the surgical management of complex pelvic tumors.

In conclusion, the management of giant uterine fibroids during cesarean section requires adherence to the principle of individualization, with key considerations including fibroid size, location, and number, as well as the parturient’s general condition and postpartum needs such as breastfeeding. However, the authors propose that for a 10-cm uterine fibroid detected in the third trimester as in this case, comprehensive preoperative evaluations should be conducted to assess the fibroid location, its relationship with the placenta, and fetal development status. Routine blood transfusion preparation and prudent intraoperative management are also recommended. Although the fibroid was identified as a broad ligament fibroid during surgery, considering the patient had no high-risk factors during pregnancy and the cesarean section incision was relatively large, myomectomy could be performed under direct visualization by experienced surgeons. Intraoperatively, meticulous anatomical identification, strict hemostasis, and avoidance of ipsilateral ureteral injury are essential. Postoperatively, routine oxytocin administration to promote uterine contraction is advisable, thereby mitigating the need for secondary surgery resulting from rapid fibroid enlargement after delivery; for fibroids not temporarily suitable for concurrent management, a rigorous postoperative follow-up mechanism needs to be established, with regular imaging monitoring of fibroid size, location, and morphological changes. Once rapid fibroid growth in a short period or obvious symptoms are detected, the possibility of malignant transformation should be vigilant, and intervention protocols should be initiated promptly to ensure early management under controllable risks. In addition, for medical institutions without the capability to manage high-difficulty giant uterine fibroids, it is recommended to promptly refer patients to higher-level medical institutions with corresponding diagnosis and treatment capabilities, ensuring that patients receive standardized treatment in a timely manner and reducing surgical risks. This case serves as a reminder that decisions regarding uterine fibroids during cesarean section should not be limited to immediate surgical safety, but also need to take long-term prognosis into account. Patients should be fully informed of the potential risks of postoperative fibroid changes, and standardized management of uterine fibroids should be implemented to minimize adverse outcomes caused by delayed treatment to the greatest extent. This paper represents a single-case report, with a relatively short follow-up period (5 months postoperatively). Given the extremely rapid growth rate of the patient’s fibroid, whether recurrence will occur after the resection of the giant uterine fibroid remains to be determined, and we will continue with long-term follow-up for this patient in the subsequent period.

## Data Availability

The original contributions presented in the study are included in the article/Supplementary Material. Further inquiries can be directed to the corresponding authors.
