# Supplementary figures and images for: Case Report: Giant uterine broad ligament fibroid
Source: Front Oncol. 2026 Jan 7;15:1712549. doi: 10.3389/fonc.2025.1712549 (PMC12819652; doi:10.3389/fonc.2025.1712549)

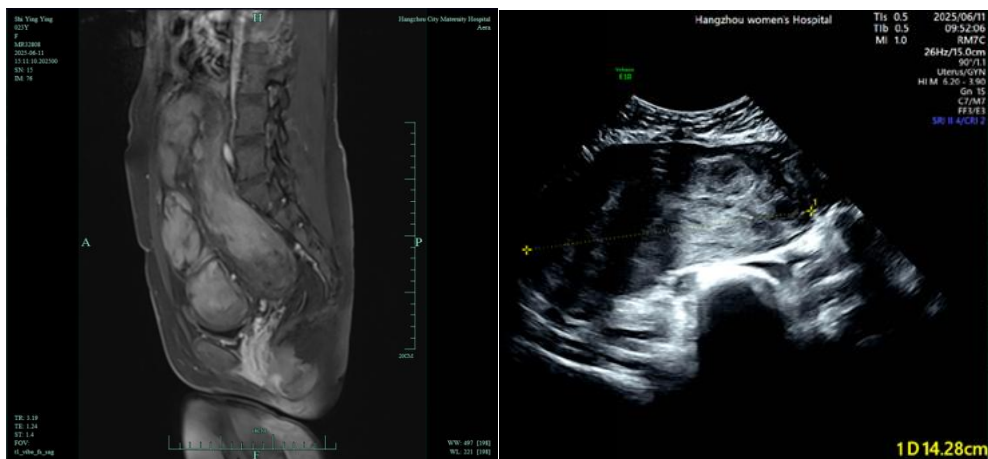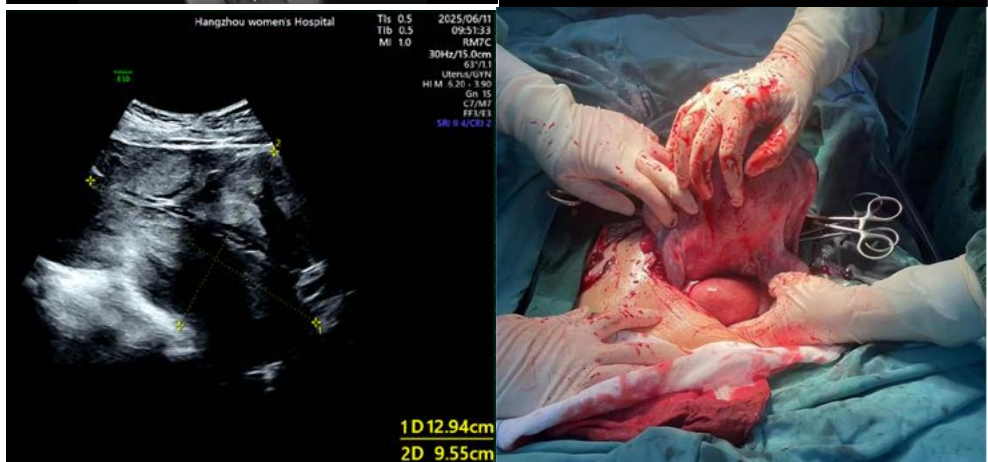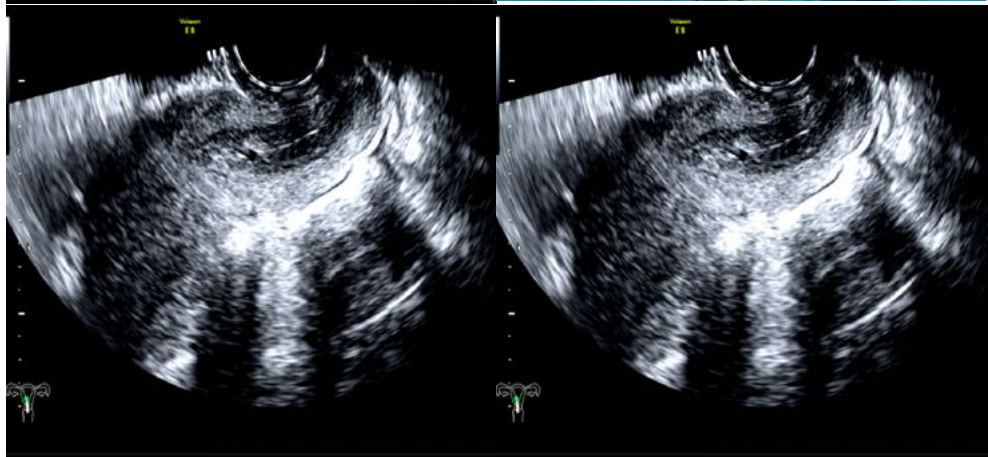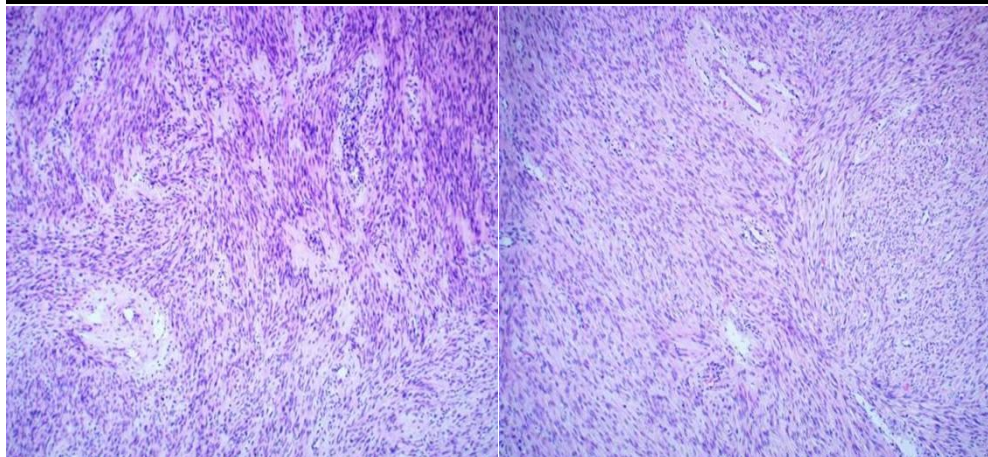

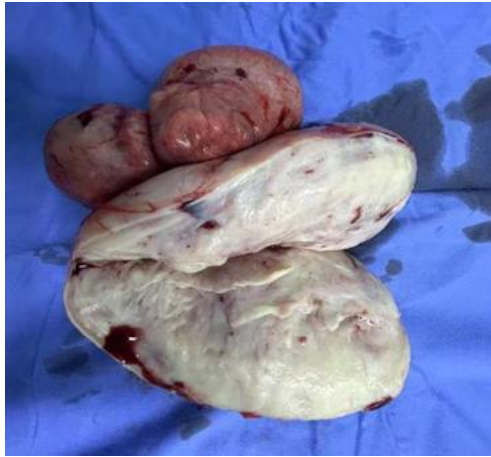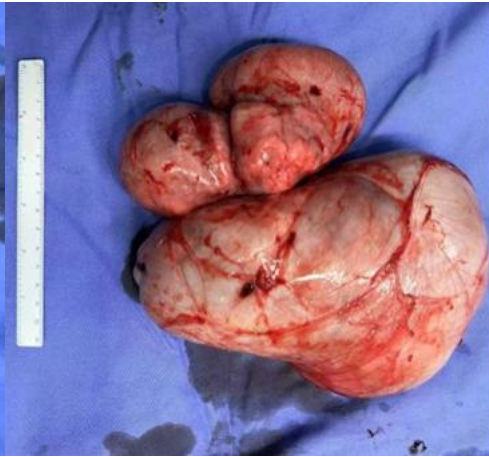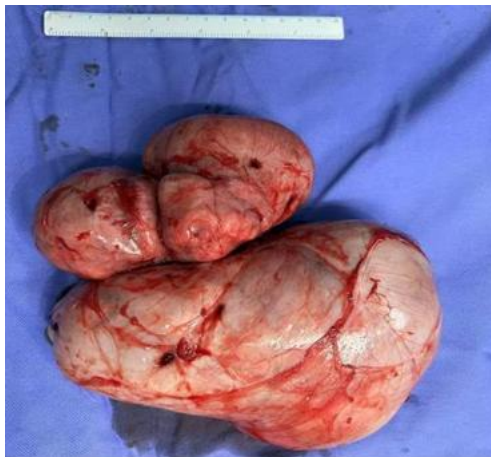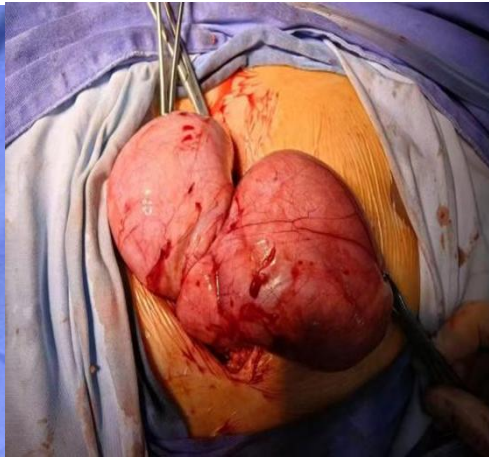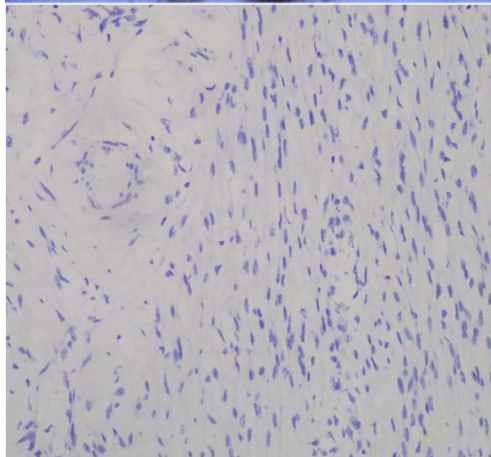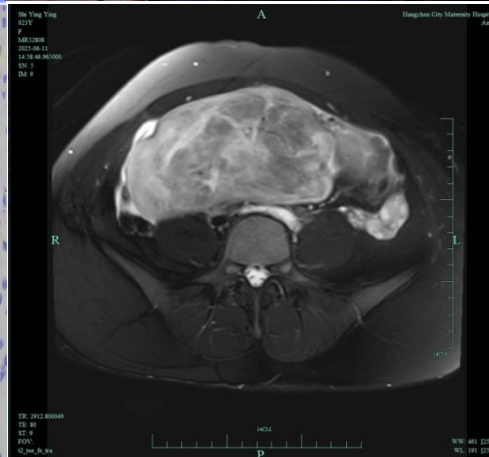

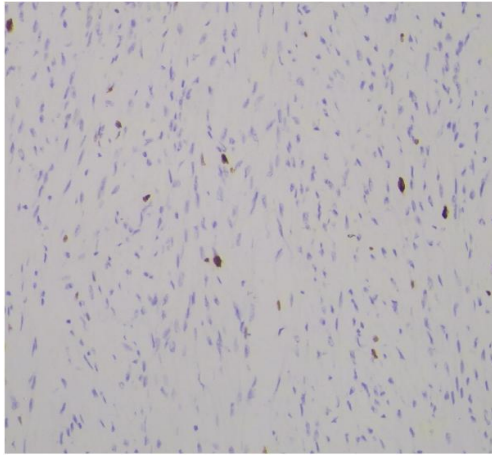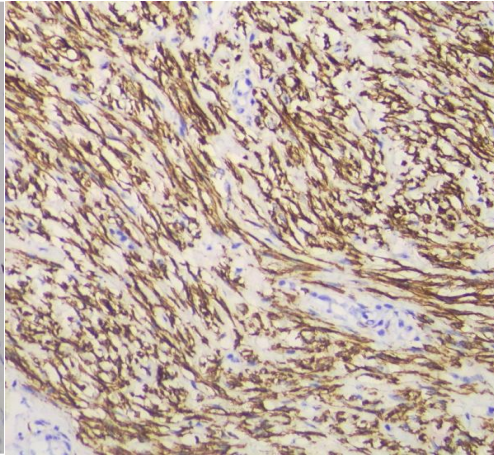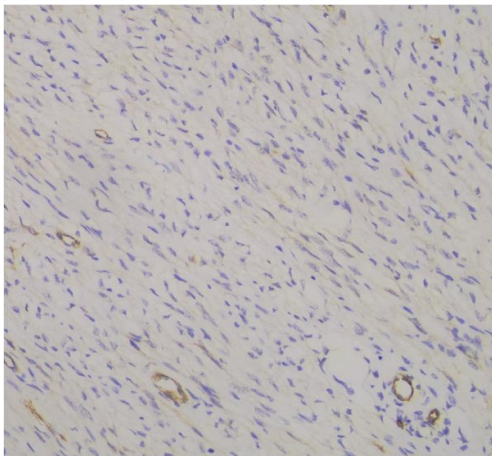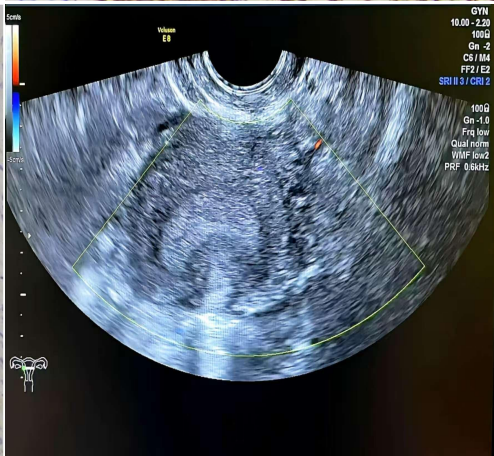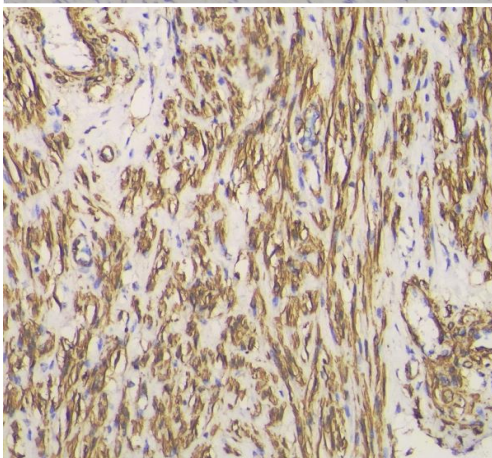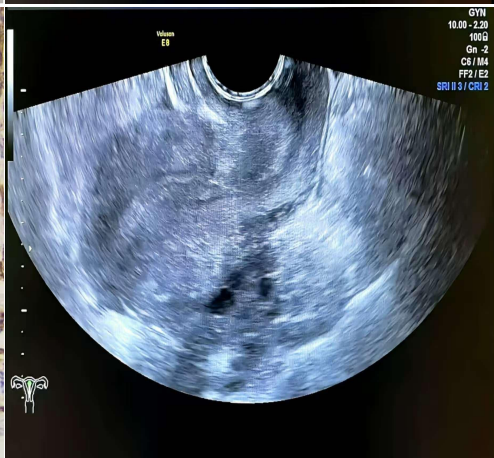

Supplement: Supplementary file 1 [file DataSheet1.pdf]
